# COVID-19 vaccine hesitancy in Ethiopia: a scoping review for equitable vaccine access

**DOI:** 10.3389/frhs.2025.1609752

**Published:** 2025-09-03

**Authors:** Senait Aleamyehu Beshah, Jibril Bashir Adem, Mosisa Bekele Degefa, Melkamu Ayalew, Yohannes Lakew, Sileshi Garoma, Elizabeth Naa Adukwei Adote, Daniel Malik Achala, Grace Njeri Muriithi, Chinyere Ojiugo Mbachu, James Akazili, Chikezie Ifeanyi, Elias Asfaw Zegeye, Chijioke O. Nwosu, John E. Ataguba

**Affiliations:** ^1^ African Health Economics and Policy Association (AfHEA), Accra, Ghana; ^2^ Health Systems Research Directorate, Ethiopian Public Health Institute, Addis Ababa, Ethiopia; ^3^ Department of Public Health, College of Health Science, Arsi University, Asella, Ethiopia; ^4^ Ethiopian Ministry of Health, Addis Ababa, Ethiopia; ^5^ Department of Community Medicine, University of Nigeria, Enugu Campus, Nigeria; ^6^ School of Public Health, C.K. Tedam University of Technology and Applied Sciences, Navrongo, Ghana; ^7^ Health System and Development Research, BCEPS University of Bergen, Bergen, Norway; ^8^ Health Systems and Development Research Group, Veritas University Abuja Nigeria Catholic Secretariat Abuja, Abuja, Nigeria; ^9^ Health Economist, Africa Centers for Disease Control and Prevention (Africa CDC), Division of Health Economics and Financing, Addis Ababa, Ethiopia; ^10^ Department of Economics and Finance, University of the Free State, Bloemfontein, South Africa; ^11^ Department of Community Health Sciences, Max Rady College of Medicine, Winnipeg, MB, Canada; ^12^ Faculty of Health Sciences, University of Manitoba, Winnipeg, Manitoba, Canada; ^13^ Partnership for Economic Policy (PEP), Nairobi, Kenya; ^14^ School of Health Systems and Public Health, University of Pretoria, Pretoria, South Africa

**Keywords:** COVID-19, hesitancy, vaccines, Ethiopia, scoping review

## Abstract

**Introduction:**

COVID-19 vaccines are crucial for preventing severe illness from the virus. Despite their effectiveness; vaccine hesitancy, unequal access, and economic disparities hinder vaccination programs across Africa, posing significant challenges in Ethiopia.

**Method:**

This scoping review followed the methodological guidelines outlined in the Joanna Briggs Institute Reviewer's and employed the Preferred Reporting Items for Systematic Reviews and Meta-Analyses - Extension for Scoping Reviews (PRISMA-ScR) checklist and explanation to ensure transparency. To analyze the data, we developed tailored search strategies for key databases [HINARI, PubMed, Cochrane, African Journals Online (AJOL), and Science Direct] and gray literature sources. These strategies combined controlled vocabulary and relevant keywords. A descriptive thematic analysis was then employed to identify and categorize the various findings within the included studies. The results are presented in a narrative format, summarizing the key themes and providing a clear and comprehensive overview of the current evidence base.

**Results and recommendations:**

A review of 34 Ethiopian studies revealed significant COVID-19 vaccine hesitancy, with rates exceeding 50% in over 40% of the studies. The lowest hesitancy was found in adults from Addis Ababa (19.1%), while the highest rates were seen among healthcare workers in Oromia (69.7%) and pregnant women in Southwest Ethiopia (68.8%). Factors contributing to vaccine hesitancy in Ethiopia include being female, having only primary education, residing in rural areas, younger age, limited knowledge about the vaccine, reduced trust in authorities, and misperceptions about the risk of the virus. To address this challenge effectively, policymakers should prioritize interventions that build public trust, enhance awareness of the vaccine's benefits, and counter misinformation.

## Introduction

## 1 Background

The COVID-19 pandemic caused by the SARS-CoV-2 virus has resulted in a significant loss of human lives worldwide and posed an unparalleled global health threat (1). The virus is contagious, spreads easily through contact and droplets. Prevention methods like distancing, hygiene, masks, and vaccines are crucial for control (2). As of June 2024, there have been over 884 million COVID-19 cases globally, with over 7 million deaths (3) and a total of 13,595,583,125 vaccine doses have been administered globally (4). While new infections slowed in Africa, the WHO African region reported over 8 million cases and 200,000 deaths (5). In Ethiopia, between January 2020 and November 2023, there were over 500,000 confirmed cases and over 7,500 deaths (6).

COVID-19 vaccines have proven highly effective in preventing symptomatic illness, with studies showing up to 95% effectiveness (7). Ethiopia began vaccinations on March 13, 2021, but as of May 27, 2023, only 59 people per 100 had received at least one dose, falling short of the global average of 74% (8). Vaccine hesitancy, the reluctance to get vaccinated despite availability, complex issue with many contributing factors. These factors range from demographics and political views to concerns about safety, lack of awareness, cultural beliefs, and socioeconomic considerations (9, 10).

COVID-19 vaccine hesitancy is a major hurdle, particularly in sub-Saharan Africa, where rates range from 14% in Ethiopia to a high of 65% in Tanzania. Several factors contribute to this hesitancy, including a history of unequal treatment in global health research, complex social and cultural beliefs, inadequate community engagement, and a general distrust in authorities (10–12).

Additionally, political influences, religious views, and a low perceived risk of contracting COVID-19 further hinder vaccine uptake in the region (13). Ethiopia faces a significant challenge in COVID-19 vaccination due to vaccine hesitancy, ranging from 14% to 69% across geographic divides, and this hesitancy stems from a combination of factors, including young age, female gender, rural residence, and limited access to information (14). To combat COVID-19 vaccine hesitancy and promote equitable access in Ethiopia, a multi-pronged approach is crucial. This includes developing culturally relevant educational materials, engaging trusted voices like religious leaders and community members, and addressing vaccine safety concerns through clear communication (15). Moreover, targeted outreach to populations with higher levels of hesitancy is crucial. Meaningful community engagement involving scientific experts plays a key role in building trust and countering misinformation (16). Additionally, addressing underlying factors contributing to hesitancy is key. Socio demographic factors like age, gender, and location, along with personal beliefs and access to accurate information, all play a role (10, 17). Vaccine hesitancy is a major hurdle to achieving equitable access to COVID-19 vaccines in Ethiopia (18–20, 15, 21). Thus, this scoping review aimed to investigates factors contributing to hesitancy and potential mitigation strategies by understanding these barriers, researchers can design interventions that promote vaccine acceptance, particularly among vulnerable populations. Increased focus on Africa-based research and development, improved stakeholder communication, and ensuring universal trust in vaccines are all crucial aspects of a multifaceted approach to achieving vaccine equity in low- and middle-income countries.

## 2 Method

### 2.1 Databases and search strategy

This scoping review was carried out following Arksey and O'Malley's methodological Framework (22). The framework consists of five stages: “(1), formulating the research questions,(2), conducting a comprehensive literature search (3), selecting relevant studies (4), extracting and organizing data and (5), synthesizing and reporting the findings.” This review adhered to the Preferred Reporting Items for Systematic Review and Meta-Analysis extension for Scoping Reviews (PRISMA-ScR) checklist (Supplementary Table-S1) (23).

The literature was identified through searches of various online databases, including HINARI, PubMed, Cochrane, African Journal Online (AJOL), and gray literature sourced through Google,Google Scholar, and other internet search engines. Medical Subject Heading (Mesh), keywords, and free text search terms were used. The search was conducted using the following terms: COVID-19 ‖ OR Coronavirus‖ OR nCoV Infection‖ OR SARS-CoV-2‖ OR COVID19,‖ MeSH terms: COVID-19 AND Vaccine‖ OR Vaccination,‖ MeSH terms: COVID-19 Vaccines AND (patterns)) AND Hesitancy OR indecision OR reluctance OR skepticism OR uncertainty AND approaches OR strategy OR method AND (barriers)) OR (factors)) OR(disfavor)) OR (dislike)) AND (equitable)) OR (Fair)) AND (timely)) OR (up-to-date)) OR (disadvantaged)) OR (underprivileged)) OR (vulnerable groups)) AND (Ethiopia). The search strategy was adapted from prior scoping or systematic reviews that assessed acceptance, uptake, and obstacles to achieving equitable and timely administration of COVID-19 and other vaccines Google Scholar, and other internet search engines. Medical Subject Heading (Mesh), keywords, and free text search terms were used. The search was conducted using the following terms: COVID-19 ‖ OR Coronavirus‖ OR nCoV Infection‖ OR SARS-CoV-2‖ OR COVID19,‖ MeSH terms: COVID-19 AND Vaccine‖ OR Vaccination, MeSH terms: COVID-19 Vaccines AND (patterns)) AND Hesitancy OR indecision OR reluctance OR skepticism OR uncertainty AND approaches OR strategy OR method AND (barriers)) OR (factors)) OR(disfavor)) OR (dislike)) AND (equitable)) OR (Fair)) AND (timely)) OR (up-to-date)) OR (disadvantaged)) OR (underprivileged)) OR (vulnerable groups)) AND (Ethiopia). The search strategy was adapted from prior scoping or systematic reviews that assessed acceptance, uptake, and obstacles to achieving equitable and timely administration of COVID-19 and other vaccines (24, 25).

### 2.2 Eligibility criteria

This scoping review sought to identify effective strategies for overcoming COVID-19 vaccine hesitancy in Ethiopia. We focused on original research published between January 2020 and early December 2023, specifically including studies conducted within Ethiopia that addressed COVID-19 vaccines. Studies solely focused on the virus itself, those from outside Ethiopia, or those not primarily focused on vaccination were excluded. Additionally, studies with incomplete or non-empirical data were omitted. This rigorous selection process ensures our review reflects the changing landscape of vaccine hesitancy in Ethiopia, offering valuable insights into current disparities in vaccine acceptance.

### 2.3 Outcome of interest

This review focused on COVID-19 vaccine hesitancy in Ethiopia, exploring the factors that contribute to it and proposing strategies to reduce hesitancy.

### 2.4 Data extraction and processing

JBA and MB independently extracted data using standardized Microsoft Excel sheet to categorize themes, summarize manuscripts, and ensure accuracy through double-checking and consensus with the primary investigator (SAB) on any discrepancies. The extraction focused on three key areas: study description (authors, year, region), methodology (design, sample size), and results (26). This research examined five key areas related to COVID-19 vaccination in Ethiopia: (1) vaccine hesitancy levels, (2) factors contributing to hesitancy (3) advocacy approaches for vaccine equity, (4) vaccine acceptance rates, and (5) barriers and solutions for equitable and timely uptake. Thematic narratives were employed to synthesize the data (26).

### 2.5 Data synthesis and analysis

Researchers employed a descriptive thematic analysis to categorize findings across the studies. This approach helped map the diverse strategies used in Ethiopia to promote equitable access to COVID-19 vaccines, reduce vaccine hesitancy, and ensure timely and equitable vaccine uptake. Key themes were then presented narratively, providing a comprehensive overview of the current evidence. Due to the heterogeneity of studies, a narrative synthesis approach was used to collect, synthesize, and map the literature.

### 2.6 Quality appraisal of the selected literature

All authors independently evaluated the quality of the studies included in the review. The Joanna Briggs Institute (JBI) framework was employed for assessing study quality (27). To evaluate the quality of evidence on factors associated with COVID-19 vaccine hesitancy and mitigation strategies in Ethiopia, we developed a scoring system. This system categorized studies as low (<49%), medium (50%–79%), or high (80%–100%) quality. To ensure the robustness and trustworthiness of our results, we implemented inter-rater reliability checks, peer reviews, and regular debriefing sessions within the research team. These iterative quality assurance measures facilitated discussions on emerging themes, refined interpretations, and ultimately enhanced the credibility of our research.

### 2.7 Ethics and dissemination

Since the data were collected from publicly available materials, this study does not require ethics approval.

### 2.8 Patient and public involvement

As this study is a scoping review, patient and public involvement (PPI) was not integrated at any stage of the research process, including the design, data collection, synthesis, reporting, or dissemination of findings.

## 3 Result

**Figure 1:**
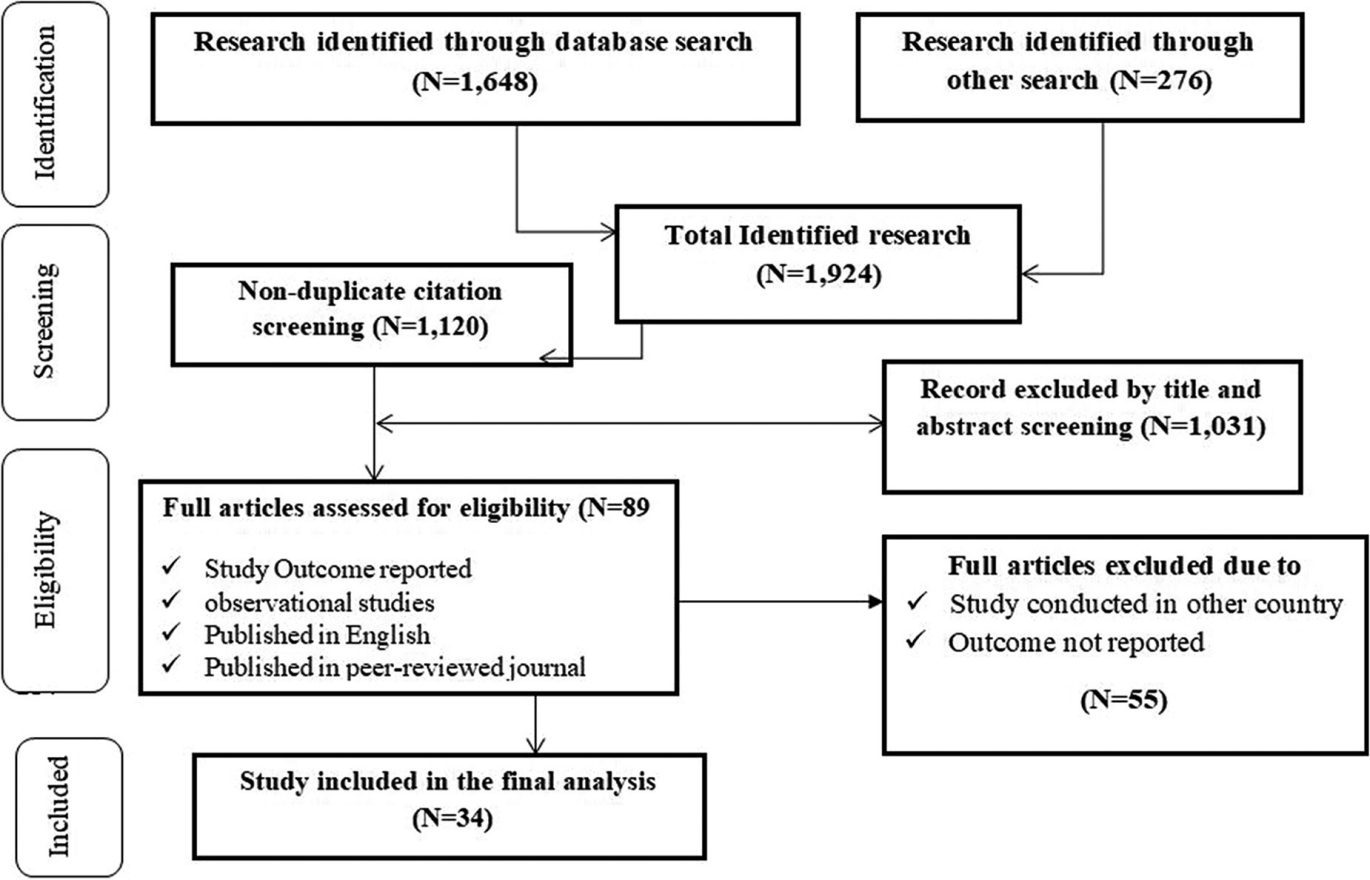
Flow chart of study.

A systematic search identified 1,924 studies on COVID-19 vaccine hesitancy and mitigation strategies in Ethiopia. After removing duplicates (*n* = 804), 1,120 articles underwent title and abstract screening. We excluded 1,031 irrelevant studies and another 55 during full-text review (non-peer-reviewed, editorials, protocols, not on hesitancy or Ethiopia, or unavailable full text). The final analysis included 34 relevant studies. See the PRISMA flow diagram (Figure. 1) for details.

### 3.1 COVID-19 vaccine hesitancy and associated factors in Ethiopia

#### 3.1.1 Characteristics of studies included in the review of COVID-19 hesitancy in Ethiopia

Our analysis of COVID-19 vaccine hesitancy in Ethiopia included 34 studies selected based on pre-defined criteria. The studies exhibited significant heterogeneity in terms of region, design, and participant demographics. The studies were conducted across different regions and administrative cities in Ethiopia, except for six studies conducted in more than one region (28–31). The other studies were conducted in a single region. These included ten studies in the Amhara region (32–38) eight in the Southern Nations Nationalities and People (11, 28)(SNNPR) (40, 41–45)region, five in the Oromia region (46–50) one in the Sidama region (51) and one in the southwestern region of Ethiopia (4). In terms of study design and participants, twenty-three institutional-based cross-sectional studies were conducted among healthcare workers (4, 30, 52–55) and patients (15, 38, 40, 56–58), university students and staff (16, 29, 44, 54, 59–61) pregnant women and lactating mothers (14, 35, 45, 59) and seven community-based cross-sectional studies were conducted among the general population (17, 30, 62–64) or the adult population (9, 38, 41). Four studies were conducted as an E-survey among the general population (47, 50) and healthcare workers (46, 53). The total number of study participants included in the review was 18,151, with individual study sample sizes ranging from 301 (14) participants to 1,361 (65). Based on the Joanna Briggs Institute quality assessment criteria, none of the studies presented a considerable risk of bias; therefore, all were included in the review (66).

### 3.2 COVID-19 vaccine hesitancy in Ethiopia

The studies included in the review reported a percentage of vaccine hesitancy ranging from 19.1% (67) to 69.7% (12) (Table 1). About 42% of the included studies reported vaccine hesitancy rates higher than 50%. The lowest hesitancy rate of 19.1% was reported among adults in Addis Ababa (67), and the highest hesitancy rate of 69.7% was reported among healthcare providers in Oromia regional state (14) and followed by pregnant women (68.8%) in south west Ethiopia (59) (Table-1).

**Table 1:** Studies included in the scoping review of COVID-19 vaccine hesitancy in Ethiopia.

Author	Title	Region	Study Design	Participants	Year	S/S	Magnitude of Hesitancy (%)	Quality Assessment
Abebe et.al	Understanding of COVID-19 Vaccine Knowledge, Attitude, Acceptance, and Determinates of COVID-19 Vaccine Acceptance Among Adult Population in Ethiopia	SNNPR	A community-based cross-sectional study	Adults	2021	492	37.4	High quality
Abetu Mehari et.al	Prevalence of COVID-19 Vaccine Hesitancy and Its Associated Factors among Chronic Disease Patients in a	Amhara	Institution-based cross-sectional study	Patientsith Chronic Disease	2023	422	49.5	High quality
Adane et.al	Knowledge, attitudes, and perceptions of COVID-19 vaccine and refusal to receive COVID-19 vaccine among healthcare workers in northeastern Ethiopia	Amhara	Institution-based cross-sectional study	Health care provider	2022	404	36	High quality
Aemro et.al	Determinants of COVID-19 vaccine hesitancy among health care workers in Amhara region referral hospitals, Northwest Ethiopia: a cross-sectional study	Amhara	E-survey	Healthcare providers	2021	440	45.9	High quality
Ahmed et.al	Intention to receive vaccine against COVID-19 and associated factors among health professionals working at public hospitals in resource limited settings	Oromia	Institution-based cross-sectional study	Healthcare providers	2021	423	46.9	High quality
Alemu et.al	COVID-19 Vaccine Hesitancy and Its Associated Factors Among Adolescents	Oromia	Institution-based cross-sectional study	Adolescents	2023	379	29	High quality
Alle et.al	Attitude and associated factors of COVID-19 vaccine acceptance among health professionals in Debre Tabor Comprehensive Specialized Hospital, North Central Ethiopia; 2021: cross-sectional study	Amhara	Institution-based cross-sectional study	Healthcare providers	2021	327	57.7	High quality
Angelo et.al	Health care workers intention to accept COVID-19 vaccine and associated factors in southwestern Ethiopia,	SNNPR	Institution-based cross-sectional study	Healthcare providers	2021	405	51.6	Medium quality
Asres et.al	COVID-19 vaccines: awareness, attitude and acceptance among undergraduate University students	Oromia	Institution-based cross-sectional study	university students	2022	387	45.7	High quality
B.Aynalem et.al	Factors associated with willingness to take COVID-19 vaccine among pregnant women at Gondar town, Northwest Ethiopia: A multicenter institution-based	Amhara	Institution-based cross-sectional study	Pregnant women	2022	510	58.6	Medium quality
Belsti et.al	Willingness of Ethiopian Population to Receive COVID-19 Vaccine	Ethiopia	E-survey	Community	2021	1,184	68.6	High quality
Bereda et.al	Explore the reasons for SARS-CoV-2 vaccine hesitancy among healthcare workers:	Oromia	Institution-based cross-sectional study	Healthcare providers	2023	422	69.7	Medium quality
Berihun et.al	Acceptance of COVID-19 Vaccine and Determinant Factors Among Patients with Chronic Disease Visiting Dessie Comprehensive Specialized Hospital, Northeastern Ethiopia	Amhara	Institution-based cross-sectional study	Patients with Chronic Disease	2021	416	40.6	High quality
Dereje et.al	COVID-19 vaccine hesitancy in Addis Ababa, Ethiopia: a mixed-method study	Addis Ababa	A community-based cross-sectional study	Adults	2022	422	19.1	High quality
Erega et.al	COVID-19 vaccine hesitancy in Ethiopia in 2021: a multicenter cross-sectional study	Amhara	Institution-based cross-sectional study	Patients attending public hospitals	2023	415	46.02	Medium quality
Hailemariam et.al	Predictors of pregnant women's intention to vaccinate against coronavirus disease 2019: A facility-based cross-sectional study in southwest Ethiopia	South West Ethiopia	Institution-based cross-sectional study	Pregnant women	2021	423	68.7	Medium quality
Handebo et.al	Determinant of intention to receive COVID-19 vaccine among school teachers in Gondar City, Northwest Ethiopia	Amhara	Institution-based cross-sectional study	School Teachers	2021	301	45.2	High
Hassen et.al	Understanding determinants of COVID-19 vaccine	Oromia	Institution-based	staffs and students	2022	358	50	Medium
Kalayou et.al	Myth and Misinformation on COVID-19 Vaccine: The Possible Impact on Vaccination Refusal Among People of Northeast Ethiopia: A Community-Based Research	Amhara	A community-based cross-sectional study	Community	2022	574	39.7	High quality
Lombebo et.al	COVID-19 Vaccine Acceptance, Attitude, Hesitancy, and Its Associated Factors among Wolaita Sodo University Students: A Mixed-Method Study	SNNPR	Institution-based cross-sectional study	University Students	2023	352	67	High quality
Mesele et.al	COVID-19 Vaccination Acceptance and Its Associated Factors in Sodo Town, Wolaita Zone, Southern Ethiopia: Cross-Sectional Study	SNNPR	A community-based cross-sectional study	Community	2021	415	54.5	High quality
Mesfin et.al	Factors Associated with Intention to Receive COVID-19 Vaccine Among HIV Positive Patients Attending ART Clinic in Southwest Ethiopia	SNNPR	Institution-based cross-sectional study	HIV Patients	2021	398	66.3	High quality
Mohammed et.al	COVID-19 vaccine hesitancy among Ethiopian healthcare workers	Addis Ababa	Institution-based cross-sectional study	Healthcare providers	2021	614	60.03	High quality
Mose et.al	COVID-19 vaccine hesitancy among medical and health science students attending Wolkite University in Ethiopia	SNNPR	Institution-based cross-sectional study	Medical and health science students	2022	420	41.2	High quality
Mose et.al	Willingness to Receive COVID-19 Vaccine and Its Determinant Factors Among Lactating Mothers in Ethiopia: A Cross-Sectional Study	SNNPR	Institution-based cross-sectional study	Lactating Mothers	2021	630	39	High quality
Muluneh et.al	COVID-19 Knowledge, Attitudes, and Vaccine Hesitancy in Ethiopia: A Community-Based Cross-Sectional Study	Ethiopia	A community-based cross-sectional study	Community	2023	1361	64.4	High
Rikitu Terefa et.al	COVID-19 Vaccine Uptake and Associated FactorAmong Health Professionals in Ethiopia	Ethiopia	E-survey	Healthcare providers	2021	522	37.9	High quality
Seboka et.al	Factors Influencing COVID-19 Vaccination Demand and Intent in Resource-Limited Settings: Based on Health Belief Model	Ethiopia	E-survey	Community	2021	1,160	35.3	High quality
Tadesse et.al	COVID-19 Vaccine Hesitancy and its Reasons in Addis Ababa, Ethiopia: A Cross-Sectional Study	Addis Ababa	A community-bas cross-sectional study	Community	2022	422	57.4	High quality
Taye et.al	Coronavirus disease 2019 vaccine acceptance and perceived barriers among university students in northeast Ethiopia: A cross-sectional study	Amhara	Institution-based cross-sectional study	university students	2021	423	30.7	High quality
Yilma et.al	COVID-19 vaccinates acceptability healthcare Workers in Ethiopia: Do we practice what we preach?	Ethiopia	Institution-based cross-sectional study	Healthcare providers	2022	1,340	25.5	High quality
Yohannes,et.al	COVID-19 vaccine hesitancy among adults in Hawass City Administration, Sidama Region, Ethiopia	Sidama	A community-based cross-sectional study	Adults	2022	622	26.5	High quality
Zewude et.al	Willingness to Take COVID-19 Vaccine Among People Most at Risk of Exposure in Southern Ethiopia	SNNPR	Institution-based cross-sectional study	Teachers, bankers and drivers	2021	384	53.9	Medium quality
Zewude et.al	Intention to Receive the Second Round of COVID-19 Vaccine Among Healthcare Workers in Eastern Ethiopia	Eastern Ethiopia	Institution-based cross-sectional study	Healthcare providers	2022	384	38.4	High quality

### 3.3 Factors associated with COVID-19 vaccine hesitancy in Ethiopia

The analysis of this review revealed the following socio-demographic factors positively linked to COVID-19 vaccines hesitancy in Ethiopia: gender (female) (14, 15, 29, 38, 42, 67) age between 18 and 29 years (14, 40) age greater than 49 years (41, 42) age less than 23 years (59), primary education level (4, 68) religion (being Muslim) (69), rural residence (14, 40) and region of residence in the Afar and Sidama regions (11). There was also a positive correlation between income level and hesitancy towards COVID-19 vaccination (42, 71) and (14, 42, 51, 72).

A lack of sufficient information regarding the vaccines short- and long-term safety (58, 70), an undefined duration of vaccine protection (71) and (59) unclear information from a public health authority (4, 14, 72), sourcing of information from social media (4, 40, 72), insufficient knowledge (48, 73) and poor attitudes toward COVID-19 vaccines (73, 74), a low (7, 14, 42, 68, 42) and poor adherence to COVID-19 prevention measures (73, 76) were the most frequently studied individual factors. Additionally, negative self-efficacy, a history of perceived and confirmed COVID-19 infection (24, 51, 58, 77) and family members who died from COVID-19 (42, 71, 42, 78, 79) were found to be important positive factors in vaccine hesitancy. The vaccine-specific determinants identified in the analysis included a lack of belief in COVID- 19 vaccine benefits (11, 42, 68, 42), lack of trust in science to produce safe and effective vaccines (11, 33, 73, 80), and concerns about vaccine safety and side effects, as well as doubts about vaccine efficacy (30, 55, 58, 60, 75). Furthermore, beliefs for using traditional medicine to prevent and cure COVID-19 have been found to be vaccine-specific factors (14, 21, 39, 81).

### 3.4 Strategies to reduce COVID-19 vaccines hesitancy in Ethiopia

This scoping review identified a variety of strategies used to reduce vaccine hesitancy and improve advocacy for equity of the vaccines in Ethiopia. Among these strategies, efforts to increase awareness about the efficacy and safety of the COVID-19 vaccines, in collaboration with various health authorities and stakeholders, to design an evidence-based strategy to reduce vaccine hesitancy, reduce concerns about vaccines side effects and vaccines effectiveness; improve trust in COVID-19 vaccines products, and change beliefs regarding acquired

being superior to vaccination, change negative concerns about the COVID-19 vaccines and change beliefs about how to treat COVID-19 with traditional remedies were the most widely shared approaches in multiple studies (14, 44, 58, 73). Additionally, improving belief in COVID-19 vaccine benefits, improving knowledge of COVID-19, educating people about the vaccine, disseminating accurate information, particularly among women, changing fear of vaccine side effects and informing healthcare administrators about the widespread prevalence of COVID-19 vaccine hesitancy in the countries were widely identified approaches by the majority of the studies (11, 14–16, 15, 38, 42, 51, 67, 75, 42, 82).

For general communities, strategies such as strengthening public education using mass media about the advantages of receiving COVID-19 vaccination, providing enough information about the COVID-19 vaccine, enabling adequate preventive practices, changing fear of vaccine side effects, changing doubts about vaccines effectiveness, improving attitudes toward the COVID-19 vaccine, and motivating users to recommend the vaccine to other people were identified in multiple studies (18, 30, 45, 54, 59, 70, 75) Similarly, for the adult population, improving people's awareness of the COVID-19 vaccine, enhancing eHealth and computer literacy, and promoting awareness of the reliability of vaccine information sources, specifically designed, culturally tailored health education materials, and high levels of engagement in the vaccination process from politicians, women community leaders (Hadha sike‖) and community figure women, religious leaders, and other community members were reported by majority of studies (11, 13, 60, 83, 84).

To reduce vaccine hesitancy and improve advocacy and equity for the vaccine among healthcare providers, improving knowledge and attitudes toward the COVID-19 vaccine and increasing trust in COVID-19 vaccine products and changing beliefs regarding acquired immunity being superior to vaccination, changing negative perceptions about the safety of COVID-19 vaccines, improving beliefs about COVID-19 vaccine benefits and providing clear information about the vaccines were reported by multiples of studies (11, 24, 29, 39, 85, 86).

(Supplementary Table S2).

## 4 Discussion

This review provides a mapping of important studies and results on COVID-19 vaccine hesitancy and predictors in Ethiopia. It also explores strategies to encourage more people to get vaccinated fairly and on-time. The quality assessment of the included studies revealed that 27 studies were rated as high quality, 7 as moderate quality, and none as poor quality based on the JBI Critical Appraisal Checklist for Analytical Cross-Sectional Studies. The majority of the research papers included were cross-sectional and examined a variety of population groups. The analysis is based on data from over 18,151 participants across all regions of Ethiopia. The magnitude of vaccine hesitancy ranged from 19.1% (12) in Addis Ababa to 69.7% to Oromia (4). Majority (58.8%) of the included studies reported vaccine hesitancy rates lower than 50%. The highest levels of vaccination hesitancy shown in several of the included studies contrasted with research conducted in other locations, such as Europe and the Americas (17, 69), China (87) Kuwait (77), and the United Kingdom (88). This may be a direct result of disparities in vaccine access, and awareness of vaccine information between countries and region in the country. Studies showed that, people in middle- or high-income groups are more likely to get vaccinated (89), and inadequate financial considerations as barriers to vaccination acceptance (90). Even while many African governments provide free vaccinations to their citizens, the reluctance of resource- constrained communities may indicate a misunderstanding about who bears the cost. Similarly, the financial burden on such communities is likely to extend beyond the vaccine itself, including transportation to vaccination centers that may not be nearby, childcare fees, and other hurdles.

This review found that women in Ethiopia were more hesitant to get vaccinated than men, leading to lower vaccination rates among women. This finding aligns with a broader review of vaccine hesitancy in Africa, which reported that men were generally more likely to be (69) accepting of the COVID-19 vaccine (75). This finding also aligns with a Canadian study on COVID-19 vaccines, where women were more hesitant than men (91). Another study found low COVID-19 vaccine acceptance among women with primary education and unemployed women (81). This aligns with a global study on immunization attitudes, which reported that unemployed women were more likely to have concerns about vaccine safety and effectiveness (85). One possible explanation for the lower vaccine acceptance among some women might be persistent misconception of COVID-19 vaccines can cause fertility problems or birth defect (92).

This misconception has caused many young women of childbearing age to hesitate about vaccination and not to consider being vaccinated. In fact, a systematic review of a sample of 703,004 pregnant women globally revealed a lower rate of vaccination among them than among the rest of the population (93).

Similarly, the results of this review showed that low education level was a factor associated with vaccine acceptance and marginally associated with high vaccine hesitancy. This finding is consistent with other studies conducted elsewhere (77, 94, 58, 95). Education plays a significant role; in a Canadian study, individuals with less than a high school education showed lower adjusted odds of wanting to vaccinate themselves against COVID-19 compared to their counterparts (96). Furthermore, Black Americans with lower educational attainment are more hesitant to accept a COVID-19 vaccine (97). A study conducted in Latin America and the Caribbean disclosed similar findings but added that lower education influences vaccine hesitancy due to the general distrust in vaccines and the robustness of conspiracy beliefs across individuals with lower education levels (98). People with lower education levels and places with higher poverty rates (99, 100), such as sub-Saharan Africa (SSA), Latin America and the Caribbean rural regions, are more likely to be skeptical of vaccines and hold conspiracy theories. In these places, health promotion initiatives lack proper coverage, increasing the danger of misinformation and hence raising resistance to immunization and community mitigation strategies. It is vital to reinforce and tailor communication techniques to these demographics.

Tailored communication improves relevance; trust, and behavioral response, ultimately enhancing public health outcomes, for example messages targeting women should consider gender roles, health literacy, and caregiving responsibilities. Community-based communication, peer networks, and maternal health platforms can be effective channels (101). Content should be accessible, empathetic, and culturally sensitive. For rural communities, leveraging trusted local leaders (e.g., religious or community figures), radio broadcasts, and mobile health units is recommended due to limited internet access and health infrastructure (102).

Visual aids and local language translations also enhance message comprehension.

Healthcare workers respond well to evidence-based, concise, and actionable information delivered via professional platforms (e.g., WhatsApp groups, clinical bulletins, or in-service training). Peer-to-peer knowledge sharing and continuous medical education can also reinforce trust and uptake (102). Additionally, this review indicated that individuals living in rural areas were more reluctant to be vaccinated. Vaccination intentions would be lower in rural areas because of the low burden of the pandemic. Similarly, these findings might be explained by the idea of a certain innate immunity, by low exposure to information, or by their poor living standards compared to other parts of the nation. This finding differs from a study in Senegal, where vaccine hesitancy and refusal were linked to living in large cities (103). Significant differences in the likelihood of immunization in more rural areas may indicate other considerations that must be addressed when planning for COVID-19 vaccination implementation.

According to recent research, indigenous dwellers and those who live in rural areas are less likely to take preventive measures like wearing masks, but are more comfortable engaging in activities that require more social interaction. Given our findings, these behaviors could have severe effects for less densely populated, conservative societies s that may be resistant to present and future preventive interventions (104).

The herd immunity threshold for the virus that causes COVID-19, SARs- CoV-2, is predicted to be 55% to 82% (103), and achieving it will necessitate coordinated efforts across the country and across all administrative areas. In preparation for their involvement in COVID-19 vaccine administration, pharmacists and other providers must consider the importance of personal and community beliefs in influencing medical recommendations and subsequent actions taken to prevent the spread of infectious diseases across all regions of the country.

Lack of sufficient information, misinformation and poor attitude regarding vaccine safety were some of the other factors associated with vaccine hesitancy. This was consistent with studies conducted in different regions of Africa (15, 93, 58, 105), Europe and America (106, 107). When it comes to the fear of adverse effects, the influence of disinformation, particularly on social media platforms, was noted in practically every research setting. Social media has a significant capacity to mediate the spread of disinformation in anti-vaccine efforts (108). Some studies (40, 72) identified social media and, to a lesser extent, traditional media as the primary sources of erroneous information dissemination. Interestingly, despite being medical students, 91% of the respondents in Kanyike and colleagues' study (108), reported getting knowledge about COVID-19 via social media rather than health specialists. Misinformation on social media exacerbated their vaccine hesitation, despite the fact that they perceived an elevated risk as a result of their engagement in COVID-19 health interventions. As these findings demonstrate, social media has enormous power in effectively disseminating information and influencing health-seeking behaviors. These influences must be central to national programs addressing vaccination reluctance. It would entail modifying the content of campaigns to appeal to individuals more strongly than the misinformation that they so readily accept. Educational strategies emphasizing vaccine safety and efficacy have been identified in the literature as an important need to overcome misinformation and enhance compliance rates (109). As Zewude and Zikarge (42) established, vaccine hesitancy can be driven by public reaction to specific vaccines. The underlying principles in these interventions should be customized to reflect the various concerns about certain vaccines. These teaching campaigns could have a greater impact if they were targeted at those individuals identified as being most anxious about vaccination. This highlights the importance of collaboration among public health organizations, healthcare professionals, and media platforms to ensure accurate information, and to deliver programs that enhance attitudes and health literacy empowering the target population to make informed decisions.

Low perceived risk of getting COVID-19, distrust in science and vaccine safety, and doubts about the vaccine's effectiveness were also among the most important factors identified by this review. People who believed vaccination wouldn't protect them or might even harm their health were more likely to be hesitant or refuse the vaccine. A number of studies indicate that worries about the safety and effectiveness of vaccines seem to play a significant role in vaccine intention (110–112). Studies emphasized the need for targeting mistrust of vaccine manufacturers and the belief that COVID-19 vaccinations will be used as targets to damage Africa (72, 73, 111, 113). Respondents were skeptical since the pharmaceutical companies were foreign and scientists from their home nations were not engaged in producing the vaccines. Further longitudinal investigations will be required to supplement the findings of this research, given the advanced stages of vaccination campaigns in many countries. In order to establish equitable widespread vaccination campaigns related to COVID-19. Therefore, good communication regarding safety and efficacy and more transparency about vaccine development and distribution, including financial aspects, should be the made a cornerstone of all other measures efforts to ensure equitable and widespread vaccination campaigns especially related to COVID-19. In addition cultural beliefs, particularly the reliance on traditional medicine, significantly influence vaccine hesitancy in Ethiopia. Traditional healers are often regarded as trusted health authorities, and many communities favor herbal and spiritual remedies over biomedical interventions, which can foster skepticism toward vaccines especially when introduced without culturally sensitive engagement (114). Mistrust in the formal health system and the perceived efficacy of traditional practices further contribute to low vaccine uptake (115). To address these challenges, policy makers' public health campaigns should collaborate with traditional healers and religious leaders to foster community trust, deliver culturally appropriate messaging, and create inclusive dialogue platforms. Utilizing local languages and storytelling can also enhance message comprehension in rural and low-literacy settings (114). Respecting cultural frameworks while promoting evidence-based practices is essential to improving vaccine acceptance and immunization coverage.

The strengths of this study include being the first to consolidate studies and key factors of COVID-19 vaccine hesitancy and its mitigation strategies in Ethiopia, aiding targeted intervention programs. The analysis provides insight into the most significant contributors to vaccine hesitancy in the country. This indicates a predominance of high-quality research in the evaluation of COVID-19 vaccine hesitancy in Ethiopia. Additionally, the strength of the study on COVID-19 vaccine acceptance in Ethiopia is the inclusion of many articles with large sample sizes, providing a comprehensive overview. However, a limitation is the lack of studies from some regions of the country such as Tigray, Afar and Somali. Additionally, there was potential selection bias due to the inclusion of only published articles but this is but this does not affect the generalizability of the study due to the high quality of the article and the adequate sample size.

## 5 Conclusion

This scoping review highlights key factors driving COVID-19 vaccine hesitancy in Ethiopia, including younger age, rural residence, low educational attainment, female gender, and individual concerns related to vaccine safety, side effects, and perceived risk. Religious and cultural beliefs further influence vaccination decisions, underscoring the need for context-specific strategies. To effectively address these challenges, policymakers should engage trusted community figures such as religious leaders and elders and implement culturally appropriate communication efforts. Recommended actions include launching targeted media campaigns via local radio and social media to dispel misinformation, training healthcare workers in risk communication and community engagement, and collaborating with grassroots organizations to deliver tailored messages to high-risk groups, particularly women, youth, and rural populations. These integrated efforts can enhance vaccine confidence and promote equitable access to immunization across the country.

## Data Availability

The original contributions presented in the study are included in the article/Supplementary Material, further inquiries can be directed to the corresponding author.

## References

[1] Shishido AA Mathew M Baddley JW. Overview of COVID-19-associated invasive fungal infection. Curr Fungal Infect Rep. (2022) 16(3):87–97. 10.1007/s12281-022-00434-0 35846240 PMC9274633

[2] Campagnano S Angelini F Fonsi GB Novelli S Drudi FM. Diagnostic imaging in COVID-19 pneumonia: a literature review. J Ultrasound. (2021) 24(4):383–95. 10.1007/s40477-021-00559-x 33590456 PMC7884066

[3] Ahmad FB Cisewski JA Anderson RN. Mortality in the United States — provisional data, 2023. MMWR Morb Mortal Wkly Rep. (2024) 73(31):677–81. 10.15585/mmwr.mm7331a1 39116025 PMC11309370

[4] Teferi G Kefale B. Coronavirus vaccine acceptance in Ethiopia: systematic review and meta-analysis. Int J Africa Nurs Sci. (2023) 19(January 2022):100598. 10.1016/j.ijans.2023.100598

[5] Dong E Ratcliff J Goyea TD Katz A Lau R Ng TK The Johns Hopkins University Center for Systems Science and Engineering COVID-19 Dashboard: data collection process, challenges faced, and lessons learned. Lancet Infect Dis. (2022) 22(12):e370–6. 10.1016/S1473-3099(22)00434-0 36057267 PMC9432867

[6] Schumacher AE Kyu HH Antony CM Aravkin AY Azhar GS Bisignano C Global age-sex-specific mortality, life expectancy, and population estimates in 204 countries and territories and 811 subnational locations, 1950–2021, and the impact of the COVID-19 pandemic: a comprehensive demographic analysis for the global burden of. Lancet. (2024) 403(10440):1989–2056. 10.1016/S0140-6736(24)00476-8 38484753 PMC11126395

[7] Tavilani A Abbasi E Kian Ara F Darini A Asefy Z. COVID-19 vaccines: current evidence and considerations. Metab Open. (2021) 12:100124. 10.1016/j.metop.2021.100124 PMC8433053 34541483

[8] WHO Coronavirus Disease (COVID-19) Dashboard. Available online at: www.Covid19.who.int

[9] Alhassan RK Aberese-Ako M Doegah PT Immurana M Dalaba MA Manyeh AK COVID-19 vaccine hesitancy among the adult population in Ghana: evidence from a pre-vaccination rollout survey. Trop Med Health. (2021) 49(1). 10.1186/s41182-021-00357-5 34915939 PMC8674411

[10] Babatope T Ilyenkova V Marais D. COVID-19 vaccine hesitancy: a systematic review of barriers to the uptake of COVID-19 vaccine among adults in Nigeria. Bull Natl Res Cent. (2023) 47(1). 10.1186/s42269-023-01017-w 36970323 PMC10028775

[11] Muluneh MD Negash K Tsegaye S Abera Y Tadesse D Abebe S COVID-19 Knowledge, attitudes, and vaccine hesitancy in Ethiopia: a community-based cross-sectional study. Vaccines (Basel). (2023) 11(4):774. 10.3390/vaccines11040774 37112686 PMC10140841

[12] Osuagwu UL Mashige KP Ovenseri-Ogbomo G Envuladu EA Abu EK Miner CA The impact of information sources on COVID-19 vaccine hesitancy and resistance in sub-saharan Africa. BMC Public Health. (2023) 23(1):1–16. 10.1186/s12889-022-14972-2 36609264 PMC9816548

[13] Ekezie W Igein B Varughese J Butt A Ukoha-Kalu BO Ikhile I Vaccination communication strategies and uptake in Africa: a systematic review. Vaccines (Basel). (2024) 12(12):1–31. 10.3390/vaccines12121333 PMC11679460 39771995

[14] Yehualashet DE Seboka BT Tesfa GA Mamo TT Yawo MN Hailegebreal S. Prevalence and determinants of COVID-19 vaccine hesitancy among the Ethiopian population: a systematic review. Risk Manag Healthc Policy. (2022) 15:1433–45. 10.2147/RMHP.S368057 35937966 PMC9346414

[15] Erega BB Ferede WY Sisay FA Tiruneh GA Ayalew AB shigign ME COVID-19 vaccine hesitancy in Ethiopia in 2021: a multicenter cross-sectional study. IJID Reg. (2023) 6(August 2022):120–4. 10.1016/j.ijregi.2022.11.006 36510492 PMC9729579

[16] Tadesse TA Antheneh A Teklu A Teshome A Alemayehu B Belayneh A COVID-19 Vaccine hesitancy and its reasons in Addis Ababa, Ethiopia: a cross-sectional study. Ethiop J Health Sci. (2022) 32(6):1061–70. 10.4314/ejhs.v32i6.2 36475258 PMC9692159

[17] Kukreti S Rifai A Padmalatha S Lin CY Yu T Ko WC Willingness to obtain COVID-19 vaccination in general population: a systematic review and meta-analysis. J Glob Health. (2022) 12:05006. 10.7189/jogh.12.05006

[18] Wollburg P Markhof Y Kanyanda S Zezza A. Assessing COVID-19 vaccine hesitancy and barriers to uptake in sub-saharan Africa. Commun Med. (2023) 3(1):1–11. 10.1038/s43856-023-00330-9 37696937 PMC10495410

[19] Hu B Yang W Bouanchaud P Chongo Y Wheeler J Chicumbe S Determinants of COVID-19 vaccine acceptance in Mozambique: the role of institutional trust. Vaccine. (2023) 41(17):2846–52. 10.1016/j.vaccine.2023.03.053 37003911 PMC10040345

[20] Dubé E Gagnon D MacDonald NE Eskola J Liang X Chaudhuri M Strategies intended to address vaccine hesitancy: review of published reviews. Vaccine. (2015) 33(34):4191–203. 10.1016/j.vaccine.2015.04.041 25896385

[21] Orangi S Pinchoff J Mwanga D Abuya T Hamaluba M Warimwe G Assessing the level and determinants of COVID-19 vaccine confidence in Kenya. Vaccines (Basel). (2021) 9(8):1–11. 10.3390/vaccines9080936 PMC8402839 34452061

[22] Arksey H O’Malley L. Scoping studies: towards a methodological framework. Int J Soc Res Methodol Theory Pract. (2005) 8(1):19–32. 10.1080/1364557032000119616

[23] Tricco AC Lillie E Zarin W O’Brien KK Colquhoun H Levac D PRISMA Extension for scoping reviews (PRISMA-ScR): checklist and explanation. Ann Intern Med. (2018) 169(7):467–73. 10.7326/M18-0850 30178033

[24] Anakpo G Mishi S. Hesitancy of COVID-19 vaccines: rapid systematic review of the measurement, predictors, and preventive strategies. Hum Vaccines Immunother. (2022) 18(5). 10.1080/21645515.2022.2074716 PMC9359354 35714274

[25] Robinson E Jones A Daly M. Since January 2020 Elsevier has created a COVID-19 resource centre with free information in English and Mandarin on the novel coronavirus COVID- 19. The COVID-19 resource centre is hosted on Elsevier Connect, the company ‘ s public news and information. (2020).

[26] Lisy K Porritt K. Narrative synthesis. Int J Evid Based Healthc. (2016) 14(4):201. 10.1097/01.XEB.0000511348.97198.8c

[27] Joanna Briggs Institute. Checklist for Systematic Reviews and Research Syntheses *[Internet]*. Adelaide, SA: Joanna Briggs Institute (2017). Available online at:. https://jbi.global/sites/default/files/2019-05/JBI_Critical_Appraisal-Checklist_for_Systematic_Reviews2017_0.pdf

[28] Assefa A Segenet Z. COVID-19 vaccine uptake and associated factors among health science university students in northeastern Ethiopia, a cross-sectional study. Hum Vaccin Immunother. (2023) 19:2208016. 10.1080/21645515.2023.2208016 37212445 PMC10208211

[29] Naidoo D Meyer-Weitz A Govender K. Factors influencing the intention and uptake of COVID-19 vaccines on the African continent: a scoping review. Vaccines (Basel). (2023) 11(4):1–33. 10.3390/vaccines11040873 PMC10146577 37112785

[30] Taruvinga T Chingono RS Marambire E Larsson L Olaru ID Sibanda S Exploring COVID-19 vaccine uptake among healthcare workers in Zimbabwe: a mixed methods study. PLOS Glob Public Heal. (2023) 3(12):1–19. 10.1371/journal.pgph.0002256 PMC10734954 38127934

[31] Endawkie A Daba C Asmare L Desye B Mawugatie TW Melak D Trends of socioeconomic and geographic inequalities in COVID-19 vaccine uptake in Ethiopia: using the WHO health equity assessment toolkit. BMC Health Serv Res. (2024) 24(1). 10.1186/s12913-024-12082-w PMC11660808 39707307

[32] Tsegaw B Kasahun F Lemma T. Since January 2020 Elsevier has created a COVID-19 resource centre with free information in English and Mandarin on the novel coronavirus COVID- 19. The COVID-19 resource centre is hosted on Elsevier Connect, the company ‘ s public news and information. (2020).

[33] Kalayou MH Awol SM. Myth and misinformation on COVID-19 vaccine: the possible impact on vaccination refusal among people of Northeast Ethiopia: a community-based research. Risk Manag Healthc Policy. (2022) 15(September):1859–68. 10.2147/RMHP.S366730 36213385 PMC9534150

[34] Handebo S Wolde M Shitu K Kassie A. Determinant of intention to receive COVID-19 vaccine among school teachers in Gondar Town, Northwest Ethiopia. PLoS One. (2021) 16(6 June):1–11. 10.1371/journal.pone.0253499 PMC8224841 34166399

[35] Aynalem ZB Bogale TW Bantie GM Ayalew AF Tamir W Feleke DG Factors associated with willingness to take COVID-19 vaccine among pregnant women at Gondar Town, Northwest Ethiopia: a multicenter institution-based cross-sectional study. PLoS One. (2022) 17(11):e0276763. 10.1371/journal.pone.0276763 36327276 PMC9632816

[36] Alle YF Oumer KE. Attitude and associated factors of COVID-19 vaccine acceptance among health professionals in Debre Tabor comprehensive Specialized Hospital, North Central Ethiopia; 2021: cross-sectional study. VirusDisease. (2021) 32(2):272–8. 10.1007/s13337-021-00708-0 34222565 PMC8231083

[37] Adane M Ademas A Kloos H. Knowledge, attitudes, and perceptions of COVID-19 vaccine and refusal to receive COVID-19 vaccine among healthcare workers in northeastern Ethiopia. BMC Public Health. (2022) 22(1):1–14. 10.1186/s12889-021-12362-8 35042476 PMC8765812

[38] Mehari EA Mekonen TG Adugnaw MT Abdela OA. Prevalence of COVID-19 vaccine hesitancy and its associated factors among chronic disease patients in a resource limited setting in Ethiopia: a cross-sectional study. Adv Public Heal. (2022) 2023(1):1776205. 10.1155/2023/1776205

[39] Shah J Abeid A Sharma K Manji S Nambafu J Korom R Perceptions and knowledge towards COVID-19 vaccine hesitancy among a subpopulation of adults in Kenya: an English survey at six healthcare facilities. Vaccines (Basel). (2022) 10(5):1–15. 10.3390/vaccines10050705 PMC9147716 35632461

[40] Kabamba Nzaji M Kabamba Ngombe L Ngoie Mwamba G Banza Ndala DB Mbidi Miema J Luhata Lungoyo C Acceptability of vaccination against COVID-19 among healthcare workers in the democratic republic of the Congo. Pragmatic Obs Res. (2020) 11:103–9. 10.2147/POR.S271096 PMC7605960 33154695

[41] Abebe H Shitu S Mose A. Understanding of COVID-19 vaccine knowledge, attitude, acceptance, and determinates of COVID-19 vaccine acceptance among adult population in Ethiopia. Infect Drug Resist. (2021) 14:2015–25. 10.2147/IDR.S312116 34103948 PMC8179743

[42] Zewude B Habtegiorgis T. Willingness to take COVID-19 vaccine among people most at risk of exposure in southern Ethiopia. Pragmatic Obs Res. (2021) 12:37–47. 10.2147/POR.S313991 PMC8166351 34079423

[43] Angelo AT Alemayehu DS Dachew AM. Health care workers intention to accept COVID-19 vaccine and associated factors in southwestern Ethiopia, 2021. PLoS One. (2021) 16(9 September):1–15. 10.1371/journal.pone.0257109 PMC8415602 34478470

[44] Mesele M. COVID-19 vaccination acceptance and its associated factors in Sodo Town, Wolaita zone, southern Ethiopia: cross-sectional study. Infect Drug Resist. (2021) 14:2361–7. 10.2147/IDR.S320771 34194232 PMC8238544

[45] Mose A. Willingness to receive COVID-19 vaccine and its determinant factors among lactating mothers in Ethiopia: a cross-sectional study. Infect Drug Resist. (2021) 14:4249–59. 10.2147/IDR.S336486 34703251 PMC8523808

[46] Fox AM Choi Y Lin L. Substantial disparities in COVID-19 vaccine uptake and unmet immunization demand in low- and middle-income countries. Health Aff. (2023) 42(12):1697–705. 10.1377/hlthaff.2023.00729 38048509

[47] Seboka BT Yehualashet DE Belay MM Kabthymer RH Ali H Hailegebreal S Factors influencing COVID-19 vaccination demand and intent in resource-limited settings: based on health belief model. Risk Manag Healthc Policy. (2021) 14:2743–56. 10.2147/RMHP.S315043 34234590 PMC8253933

[48] Kunyenje CA Chirwa GC Mboma SM Ng’ambi W Mnjowe E Nkhoma D COVID-19 vaccine inequity in African low-income countries. Front Public Heal. (2023) 11:1087662. 10.3389/fpubh.2023.1087662 PMC10025287 36950103

[49] Momentum U Immunization R. Covid-19 Vaccination Integration Assessment.

[50] Sanny JA. Africans are Split on COVID-19 Vaccination; Don't Trust Government to Ensure Vaccine Safety (Pan-Africa Profile No. 553). Accra: Afrobarometer (2022). p. 1–27.

[51] Yohannes S Alemayehu A Woldesenbet YM Tadele T Dangiso D Birhanu M COVID-19 vaccine hesitancy among adults in hawassa city administration, sidama region, Ethiopia: a community-based study. Front Public Heal. (2023) 11:1122418. 10.3389/fpubh.2023.1122418 PMC10017993 36935692

[52] Mehari EA Mekonen TG Adugnaw MT Abdela OA. Prevalence of COVID-19 vaccine hesitancy and its associated factors among chronic disease patients in a resource limited setting in Ethiopia: a cross-sectional study. Adv Public Health. (2022) 2023(1):1776205. 10.1155/2023/1776205

[53] Tebabere M Tesfanesh L Mulualem S Moges S Abayneh S Temesgen G COVID-19 vaccine acceptance among health care professionals in Ethiopia: a systematic review and meta-analysis. Hum Vaccin Immunother. (2023) 19:2188854. 10.1080/21645515.2023.2188854 36949629 PMC10072108

[54] Wiysonge CS Alobwede SM de Marie C Katoto P Kidzeru EB Lumngwena EN Cooper S COVID-19 vaccine acceptance and hesitancy among healthcare workers in South Africa. Expert Rev Vaccines. (2022) 21(4):549–59. 10.1080/14760584.2022.2023355 34990311

[55] Solís Arce JS Warren SS Meriggi NF Scacco A McMurry N Voors M COVID-19 vaccine acceptance and hesitancy in low- and middle-income countries. Nat Med. (2021) 27(8):1385–94. 10.1038/s41591-021-01454-y 34272499 PMC8363502

[56] Hu D Kong Y Li W Han Q Zhang X Zhu LX Frontline nurses’ burnout, anxiety, depression, and fear statuses and their associated factors during the COVID-19 outbreak in Wuhan, China: a large-scale cross-sectional study. EClinicalMedicine. (2020) 24. 10.1016/j.eclinm.2020.100424 PMC7320259 32766539

[57] Vagni M Giostra V Maiorano T Santaniello G Pajardi D. Personal accomplishment and hardiness in reducing emergency stress and burnout among COVID-19 emergency workers. Sustain. (2020) 12(21):9071. 10.3390/su12219071

[58] Berihun G Walle Z Berhanu L Teshome D. Acceptance of COVID-19 vaccine and determinant factors among patients with chronic disease visiting dessie comprehensive specialized hospital, northeastern Ethiopia. Patient Prefer Adherence. (2021) 15:1795–805. 10.2147/PPA.S324564 34429591 PMC8380286

[59] Sahile AT Gizaw GD Mgutshini T Gebremariam ZM Bekele GE. COVID-19 Vaccine acceptance level in Ethiopia: a systematic review and meta-analysis. Can J Infect Dis Med Microbiol. (2022) 2022. 10.1155/2022/2313367 36061634 PMC9436617

[60] Eguavoen A Larson H Chinye-Nwoko F Ojeniyi T. Reducing COVID-19 vaccine hesitancy and improving vaccine uptake in Nigeria. J Public Health Africa. (2023) 14(5). 10.4081/jphia.2023.2290 PMC10365642 37492424

[61] Jalili M Niroomand M Hadavand F Zeinali K Fotouhi A. Burnout among healthcare professionals during COVID-19 pandemic: a cross-sectional study. Int Arch Occup Environ Health. (2021). 10.1007/s00420-021-01695-x 33864490 PMC8052946

[62] Tariq S Hamza M Muazzam A Ahmer A Mumtaz MT Ahmad S. COVID-19 a brief overview. Brill Res Artif Intell. (2022) 2(3):107–13. 10.47709/brilliance.v2i3.1601

[63] Dula J Mulhanga A Nhanombe A Cumbi L Júnior A Gwatsvaira J COVID-19 vaccine acceptability and its determinants in Mozambique: an online survey. Vaccines (Basel). (2021) 9(8):1–10. 10.3390/vaccines9080828 PMC8402577 34451953

[64] Masresha B Ruiz MAS Atuhebwe P Mihigo R. The first year of COVID-19 vaccine roll-out in Africa: challenges and lessons learned. Pan Afr Med J. (2022) 41(Supp 2):1–10. 10.11604/pamj.supp.2022.41.2.33686 PMC9474932 36159028

[65] Bokolo M Mansouri A Michaud S. Perceptions and hesitancy towards the COVID-19 vaccination campaign among three vulnerable populations in the Democratic Republic of the Congo: a qualitative study. Niger Med J. (2024) 65(1):40–55. 10.60787/nmj-v65i1-450 39006177 PMC11238165

[66] Dos Santos WM Secoli SR de Araújo Püschel VA. The joanna briggs institute approach for systematic reviews. Rev Lat Am Enfermagem. (2018) 26:e3074. 10.1590/1518-8345.2885.3074 30462787 PMC6248737

[67] Dereje N Tesfaye A Tamene B Alemeshet D Abe H Tesfa N COVID-19 vaccine hesitancy in Addis Ababa, Ethiopia: a mixed-method study. BMJ Open. (2022) 12(5):e052432. 10.1136/bmjopen-2021-052432 35636790 PMC9152622

[68] Zewude B Belachew A. Intention to receive the second round of COVID-19 vaccine among healthcare workers in eastern Ethiopia. Infect Drug Resist. (2021) 14(July):3071–82. 10.2147/IDR.S326055 34408451 PMC8364848

[69] Belsti Y Gela YY Dagnew YAB Getnet M Seid MA Diress M Willingness of Ethiopian population to receive COVID-19 vaccine. J Multidiscip Healthc. (2021) 14:1233–43. 10.2147/JMDH.S312637 34093019 PMC8169050

[70] Asres F Umeta B. COVID-19 vaccines: awareness, attitude and acceptance among undergraduate university students. J Pharm Policy Pract. (2022) 15(1):1–7. 10.1186/s40545-021-00397-6 35473953 PMC9040694

[71] Hassen HD Welde M Menebo MM. Understanding determinants of COVID-19 vaccine hesitancy; an emphasis on the role of religious affiliation and individual’s reliance on traditional remedy. BMC Public Health. (2022) 22(1):1–11. 10.1186/s12889-022-13485-2 35672720 PMC9172606

[72] Gonçalves BA de Souza Amorim Matos CC dos Santos Ferreira JV Itagyba RF Moço VR Couto MT. COVID-19 vaccine hesitancy in Latin America and Africa: a scoping review. Cad Saude Publica. (2023) 39(8):e00041423. 10.1590/0102-311xpt041423 37556613 PMC10494688

[73] Liga AD Jabir YN Bacha RH. COVID-19 vaccine acceptance and adherence to non-pharmaceutical interventions among employees of public transportations company in Addis Ababa, Ethiopia. Hum Vaccines Immunother. (2023) 19(1):1–7. 10.1080/21645515.2023.2184759 PMC10026860 36880671

[74] Bereda G. Explore the reasons for SARS-CoV-2 vaccine hesitancy among healthcare workers: a cross- sectional study. Ann Med Surg. (2023) 85(6):2443–50. 10.1097/MS9.0000000000000628 PMC10289542 37363532

[75] Ackah BBB Woo M Stallwood L Fazal ZA Okpani A Ukah UV COVID-19 vaccine hesitancy in Africa: a scoping review. Glob Heal Res Policy. (2022) 7(1):1–20. 10.1186/s41256-022-00255-1 PMC9294808 35850783

[76] Reddy KP Shebl FM Foote JHA Harling G Scott JA Panella C Cost-effectiveness of public health strategies for COVID-19 epidemic control in South Africa: a microsimulation modelling study. Lancet Glob Heal. (2021) 9(2):e120–9. 10.1016/S2214-109X(20)30452-6 PMC7834260 33188729

[77] Alqudeimat Y Alenezi D Alhajri B Alfouzan H Almokhaizeem Z Altamimi S Acceptance of a COVID-19 vaccine and its related determinants among the general adult population in Kuwait. Med Princ Pract. (2021) 30(3):262–71. 10.1159/000514636 33486492 PMC8089409

[78] Zewudie A Regasa T Kebede O Abebe L Feyissa D Ejata F Healthcare professionals’ willingness and preparedness to work during COVID-19 in selected hospitals of southwest Ethiopia. Risk Manag Healthc Policy. (2021) 14:391–404. 10.2147/RMHP.S289343 33568957 PMC7868776

[79] Terefa DR Shama AT Feyisa BR Desisa AE Geta ET Cheme MC COVID-19 vaccine uptake and associated factors among health professionals in Ethiopia. Infect Drug Resist. (2021) 14:5531. 10.2147/IDR.S344647 34984008 PMC8702775

[80] Getachew Asmare A Gizachew AK Amanuel YG Natnael AG Molalegn MG Endeshaw CA Knowledge and attitude towards COVID-19 vaccine in Ethiopia: systematic review and meta-analysis. Hum Vaccin Immunother. (2023) 19:2179224. 10.1080/21645515.2023.2179224 36882983 PMC10026859

[81] Bhattacharya O Siddiquea BN Shetty A Afroz A Billah B. COVID-19 vaccine hesitancy among pregnant women: a systematic review and meta-analysis. BMJ Open. (2022) 12(8):1–6. 10.1136/bmjopen-2022-061477 PMC9393853 35981769

[82] Abubakari SW Workneh F Asante KP Hemler EC Madzorera I Wang D Determinants of COVID-19 vaccine readiness and hesitancy among adults in sub-saharan Africa. PLOS Glob Public Heal. (2023) 3(7):e0000713. 10.1371/journal.pgph.0000713 PMC10348558 37450441

[83] Lawal L Aminu Bello M Murwira T Avoka C Yusuf Ma’aruf S Harrison Omonhinmin I Low coverage of COVID-19 vaccines in Africa: current evidence and the way forward. Hum Vaccines Immunother. (2022) 18(1):1–5. 10.1080/21645515.2022.2034457 PMC9009957 35240908

[84] Nigus M Zelalem M Abraham K Shiferaw A Admassu M Masresha B. Implementing nationwide measles supplemental immunization activities in Ethiopia in the context of COVID-19: process and lessons learnt. Pan Afr Med J. (2020) 37(Supp 1):36. 10.11604/pamj.supp.2020.37.1.26614 33456660 PMC7796832

[85] Descamps A Launay O Bonnet C Blondel B. Seasonal influenza vaccine uptake and vaccine refusal among pregnant women in France: results from a national survey. Hum Vaccines Immunother. (2020) 16(5):1093–100. 10.1080/21645515.2019.1688035 PMC7227616 31725346

[86] Boro E Stoll B. Barriers to COVID-19 health products in low-and middle-income countries during the COVID-19 pandemic: a rapid systematic review and evidence synthesis. Front Public Heal. (2022) 10:928065. 10.3389/fpubh.2022.928065 PMC9354133 35937225

[87] Lin HH Ezzati M Murray M. Tobacco smoke, indoor air pollution and tuberculosis: a systematic review and meta-analysis. PLoS Med. (2007) 4(1):0173–89. 10.1371/journal.pmed.0040020 PMC1769410 17227135

[88] Williams L Gallant AJ Rasmussen S Brown Nicholls LA Cogan N Deakin K Towards intervention development to increase the uptake of COVID-19 vaccination among those at high risk: outlining evidence-based and theoretically informed future intervention content. Br J Health Psychol. (2020) 25(4):1039–54. 10.1111/bjhp.12468 32889759

[89] Ditekemena JD Nkamba DM Mutwadi A Mavoko HM Fodjo JNS Luhata C COVID-19 vaccine acceptance in the democratic Republic of Congo: a cross-sectional survey. Vaccines (Basel). (2021) 9(2):1–11. 10.3390/vaccines9020153 PMC7917589 33672938

[90] Mustapha M Lawal BK Sha’aban A Jatau AI Wada AS Bala AA Factors associated with acceptance of COVID-19 vaccine among university health sciences students in northwest Nigeria. PLoS One. (2021) 16(11 November):1–15. 10.1371/journal.pone.0260672 PMC8629299 34843594

[91] Cénat JM Noorishad PG Moshirian Farahi SMM Darius WP Mesbahi El Aouame A Onesi O Prevalence and factors related to COVID-19 vaccine hesitancy and unwillingness in Canada: a systematic review and meta-analysis. J Med Virol. (2023) 95(1). 10.1002/jmv.28156 PMC9538578 36114154

[92] Schaler L Wingfield M. COVID-19 vaccine—can it affect fertility? Ir J Med Sci. (2022) 191(5):2185–7. 10.1007/s11845-021-02807-9 34651258 PMC8516490

[93] Galanis P Vraka I Siskou O Konstantakopoulou O Katsiroumpa A Kaitelidou D. Uptake of COVID-19 vaccines among pregnant women: a systematic review and meta-analysis. Vaccines (Basel). (2022) 10(5):766. 10.3390/vaccines10050766 35632521 PMC9145279

[94] Bhattacharya O Siddiquea BN Shetty A Afroz A Billah B. COVID-19 vaccine hesitancy among pregnant women: a systematic review and meta-analysis. BMJ Open. (2022) 12(8):e061477. 10.1136/bmjopen-2022-061477 35981769 PMC9393853

[95] Patwary MM Alam MA Bardhan M Disha AS Haque MZ Billah SM COVID-19 Vaccine acceptance among low- and lower-middle-income countries: a rapid systematic review and meta-analysis. Vaccines (Basel). (2022) 10(3):427. 10.3390/vaccines10030427 35335059 PMC8950670

[96] Ogilvie GS Gordon S Smith LW Albert A Racey CS Booth A Intention to receive a COVID-19 vaccine: results from a population-based survey in Canada. BMC Public Health. (2021) 21(1):1–14. 10.1186/s12889-021-11098-9 34051770 PMC8164402

[97] Gatwood J McKnight M Fiscus M Hohmeier KC Chisholm-Burns M. Factors influencing likelihood of COVID-19 vaccination: a survey of Tennessee adults. Am J Heal Pharm AJHP Off J Am Soc Heal Pharm. (2021) 78(10):879–89. 10.1093/ajhp/zxab099 PMC7989652 33954426

[98] Hernandez AV Benites-zapata VA. Since January 2020 Elsevier has created a COVID-19 resource centre with free information in English and Mandarin on the novel coronavirus COVID- 19. The COVID-19 resource centre is hosted on Elsevier Connect, the company ‘ s public news and information. (2020).

[99] Atun R de Andrade LOM Almeida G Cotlear D Dmytraczenko T Frenz P Health-system reform and universal health coverage in Latin America. Lancet (London, England). (2015) 385(9974):1230–47. 10.1016/S0140-6736(14)61646-9 25458725

[100] French J Deshpande S Evans W Obregon R. Key guidelines in developing a pre-emptive COVID-19 vaccination uptake promotion strategy. Int J Environ Res Public Health. (2020) 17(16):1–14. 10.3390/ijerph17165893 PMC7459701 32823775

[101] Sampson M Xu W Prabhu S. Tailoring perinatal health communication: centering the voices of mothers at risk for maternal mortality and morbidity. Int J Environ Res Public Health. (2023) 20(1):186. 10.3390/ijerph20010186 PMC9819297 36612508

[102] Asfaw S Morankar S Abera M Mamo A Abebe L Bergen N Talking health: trusted health messengers and effective ways of delivering health messages for rural mothers in southwest Ethiopia. Arch Public Heal. (2019) 77:8. 10.1186/s13690-019-0334-4 PMC6383212 30828451

[103] Ba MF Faye A Kane B Diallo AI Junot A Gaye I Factors associated with COVID-19 vaccine hesitancy in Senegal: a mixed study. Hum Vaccin Immunother. (2022) 18(5):2060020. 10.1080/21645515.2022.2060020 35543616 PMC9897646

[104] Samore T Fessler DMT Sparks AM Holbrook C Aarøe L Baeza CG Greater traditionalism predicts COVID-19 precautionary behaviors across 27 societies. Sci Rep. (2023) 13(1):1–13. 10.1038/s41598-023-29655-0 37041216 PMC10090070

[105] Dinga JN Njoh AA Gamua SD Muki SE Titanji VPK. Factors driving COVID-19 vaccine hesitancy in Cameroon and their implications for Africa: a comparison of two cross-sectional studies conducted 19 months apart in 2020 and 2022. Vaccines (Basel). (2022) 10(9):1–14. 10.3390/vaccines10091401 PMC9503216 36146479

[106] Khubchandani J Macias Y. COVID-19 vaccination hesitancy in hispanics and African-Americans: a review and recommendations for practice. Brain, Behav Immun Heal. (2021) 15(May):100277. 10.1016/j.bbih.2021.100277 PMC8137342 34036287

[107] Yasmin F Najeeb H Moeed A Naeem U Asghar MS Chughtai NU COVID-19 Vaccine hesitancy in the United States: a systematic review. Front Public Heal. (2021) 9:770985. 10.3389/fpubh.2021.770985 PMC8650625 34888288

[108] Assegu YB Bidu KT Mosa AA Bekele EA. Covid 19 vaccination implementation among preparatory school students in Akaki Kality sub city, Addis Ababa, Ethiopia. WJBPHS. (2024) 18(1):257–70. 10.30574/wjbphs.2024.18.1.0203

[109] Dror AA Eisenbach N Taiber S Morozov NG Mizrachi M Zigron A Vaccine hesitancy: the next challenge in the fight against COVID-19. Eur J Epidemiol. (2020) 35(8):775–9. 10.1007/s10654-020-00671-y 32785815 PMC8851308

[110] Al-Jayyousi GF Sherbash MAM Ali LAM El-Heneidy A Alhussaini NWZ Elhassan MEA Factors influencing public attitudes towards COVID-19 vaccination: a scoping review informed by the socio-ecological model. Vaccines (Basel). (2021) 9(6):1–27. 10.3390/vaccines9060548 PMC8225013 34073757

[111] Alabdulla M Reagu SM Al-Khal A Elzain M Jones RM. COVID-19 vaccine hesitancy and attitudes in Qatar: a national cross-sectional survey of a migrant-majority population. Influenza Other Respi Viruses. (2021) 15(3):361–70. 10.1111/irv.12847 PMC8014858 33605010

[112] Bell S Clarke R Mounier-Jack S Walker JL Paterson P. Parents’ and guardians’ views on the acceptability of a future COVID-19 vaccine: a multi-methods study in England. Vaccine. (2020) 38(49):7789–98. 10.1016/j.vaccine.2020.10.027 33109389 PMC7569401

[113] Agyekum MW Afrifa-Anane GF Kyei-Arthur F Addo B. Acceptability of COVID-19 vaccination among health care workers in Ghana. Adv Public Heal. (2021) 2021(1):9998176. 10.1155/2021/9998176

[114] Kassaye K Amberbir A Getachew B Mussema Y. A historical overview of traditional medicine practices and policy in Ethiopia. Ethiop J Heal Dev. (2007) 20(2). 10.4314/ejhd.v20i2.10023

[115] Stoop N Hirvonen K Maystadt JF. Institutional mistrust and child vaccination coverage in Africa. BMJ Glob Heal. (2021) 6(4):e004595. 10.1136/bmjgh-2020-004595 PMC8094341 33926893

