# Supplementary material for: COVID-19 vaccine hesitancy in Ethiopia: a scoping review for equitable vaccine access
Source: Front Health Serv. 2025 Sep 3;5:1609752. doi: 10.3389/frhs.2025.1609752 (PMC12444891; doi:10.3389/frhs.2025.1609752)
Supplement: Supplementary file 1 [file Table1.docx]

**Supplementary table 2: Approaches used to reduce vaccine hesitancy and improve vaccine acceptance in Ethiopia**

| **Titles** | **Study design** | **Region** | **Participant** | **Year** | **Sample size** | **Approaches to reduce COVID-19 vaccine hesitancy** |
| --- | --- | --- | --- | --- | --- | --- |
| COVID-19 vaccine hesitancy among Ethiopian healthcare workers([28](#_ENREF_28)) | Institutional-based cross-sectional study | Addis Ababa | Healthcare workers | 2021 | 614 | Changing belief regarding acquired immunity is superior than vaccination  Improving trust in COVID-19 vaccine product  Improving trust in the Ministry of Health  Changing the negative concern on COVID-19 vaccine  Changing negative perception on the safety of COVID-19 vaccine  Improving believe in COVID-19 vaccine benefits |
| COVID-19 vaccine hesitancy in Addis Ababa, Ethiopia: a mixed-method study([22](#_ENREF_22)) | Concurrent mixed-method | Addis Ababa | Adult | 2022 | 422 | Improving information about COVID-19 vaccine  Solving fear effectiveness COVID-19 vaccine  Changing perception on side effects COVID-19 vaccine  Improving attitude toward COVID-19 and its preventive measures |
| COVID-19 Knowledge, Attitudes, and Vaccine Hesitancy in Ethiopia([29](#_ENREF_29)) | A Community-Based Cross-Sectional Study | Ethiopia | Community | 2023 | 1361 | Enabling adequate preventive practices |
| Understanding determinants of COVID-19 vaccine hesitancy ; an emphasis on the role of religious affiliation and individual's reliance on traditional remedy([60](#_ENREF_60)) | Cross-sectional survey | Jimma University | Staffs and students | 2022 | 358 | Reducing concern toward vaccine side effect and vaccine effectiveness  Changing belief to treat COVID-19 with traditional remedies |
| COVID-19 Vaccine Hesitancy and Its Associated Factors among Adolescents([20](#_ENREF_20)) | Institutional-based cross-sectional study | Jimma | Adult | 2021 | 379 | Improving knowledge of COVID-19 disease |
| Determinants of COVID-19 vaccine hesitancy among health care workers in Amhara region referral hospitals, Northwest Ethiopia([54](#_ENREF_54)) | Cross-sectional survey | Amhara | Healthcare workers healthcare workers | 2021 | 418 | Provision of clear information about the vaccine |
| Prevalence and Determinants of COVID-19 Vaccine Hesitancy Among the Ethiopian Population([52](#_ENREF_52)) | Systematic Review | Ethiopia | Community | 2022 | 9897 | Healthcare managers should acknowledge the widespread prevalence of COVID-19 vaccine hesitancy in Ethiopian regions |
| COVID-19 Vaccine Hesitancy and its Reasons in Addis Ababa, Ethiopia([30](#_ENREF_30)) | Cross-sectional survey | Addis Ababa | Community | 2022 | 422 | Changing fear of vaccine side effects  Changing doubt about its effectiveness  Providing enough information about the COVID-19 vaccine |
| Prevalence of COVID-19 Vaccine Hesitancy and Its Associated Factors among Chronic Disease Patients in a Resource Limited Setting in Ethiopia([52](#_ENREF_52)) | Institutional-based cross-sectional study | Northwest Ethiopia | Chronic patient | 2021 | 422 | Delivering more information about the COVID-19 vaccine’s safety and efficacy  Disseminating accurate information, particularly among women  Educating people about the vaccine |
| COVID-19 vaccine hesitancy among adults in Hawassa City Administration, Sidama Region, Ethiopia([68](#_ENREF_68)) | Community-Based Cross-Sectional Study | Sidama | Adults | 2023 | 622 | Improving people's awareness COVID-19 vaccine’s  Focus interventions on the identified risk factors for vaccine hesitancy |
| Myth and Misinformation on COVID-19 Vaccine: The Possible Impact on Vaccination Refusal Among People of Northeast Ethiopia: A Community-Based Research([58](#_ENREF_58)) | Community-Based Cross-Sectional Study | Northeast Ethiopia | Adults | 2022 | 574 | Enhancing community’s eHealth and computer literacy  Promoting awareness of the reliability of vaccine information sources |
| COVID-19 Knowledge, Attitudes, and Vaccine Hesitancy in Ethiopia: A Community-Based Cross-Sectional Study([29](#_ENREF_29)) | Community-Based Cross-Sectional Study | Amhara | adults | 2023 | 1361 | Specifically designed, culturally tailored health education materials  High level of engagement from politicians, religious leaders, and other community members |
| COVID-19 vaccine hesitancy , adverse events following immunization, and associated factors among the Ethiopian general population during the early phase of the COVID-19 vaccination program([112](#_ENREF_112)) | Community-Based Cross-Sectional Study | Ethiopia | Community | 2021 | 853 | Improving attitudes toward the COVID-19 vaccine  Motivating users to recommend the vaccine to other people |
| COVID-19 vaccine acceptance and its determinants among residents of Ambo Town, West Shewa, Oromia Region, Ethiopia: cross-sectional survey([113](#_ENREF_113)) | Community-Based Cross-Sectional Study | Ambo Town | Community | 2022 | 385 | Strengthen public education using mass media about the advantages of getting the COVID-19 vaccination |
| COVID-19 vaccine hesitancy among health professionals in a tertiary care center at the University of Gondar Specialized Hospital, Ethiopia: A cross-sectional study([114](#_ENREF_114)) | Institutional-based cross-sectional study | University of Gondar Specialized Hospital. | Health professionals | 2022 | 319 | COVID-19 vaccination programs should be redesigned to remove barriers to vaccine acceptance  Improving Knowledge and attitude toward the COVID-19 vaccine |
| COVID-19 Vaccine Acceptance, Attitude, Hesitancy , and Its Associated Factors among Wolaita Sodo University Students: A Mixed-Method Study([27](#_ENREF_27)) | Institutional-based cross-sectional study | Wolaita Sodo University Students | Health science students | 2023 | 352 | Design an evidence-based strategy to reduce vaccine Hesitancy |
